# Prevalence of Osteoporosis and Osteopenia in Patients With Type 2 Diabetes at King Abdulaziz University Hospital: A Retrospective Analysis

**DOI:** 10.7759/cureus.77624

**Published:** 2025-01-18

**Authors:** Mohammad M Bassi, Ibrahim R Halawani, Hashim A Alshehri, Faisal S Alyahya, Mohannad Y Alhindi, Rayan A Alamri, Mohammed A Alamri, Abdullah A Altuwairqi

**Affiliations:** 1 Faculty of Medicine, King Abdulaziz University, Jeddah, SAU; 2 Orthopedic Surgery, King Abdulaziz University, Jeddah, SAU

**Keywords:** body mass index, bone mineral density, osteopenia, osteoporosis, type 2 diabetes

## Abstract

Introduction

Osteoporosis mainly affects postmenopausal women and is characterized by decreasing bone mass and an increased risk of fracture. As populations age, it becomes more common and is frequently missed until fractures happen. Simultaneously, there is conflicting evidence about the relationship between bone health and diabetes, a chronic metabolic illness, with varying effects on the skeletal system. In light of the rising incidence of both disorders, this study examines the relationship between diabetes and osteoporosis/osteopenia in patients at King Abdulaziz University Hospital (KAUH), Jeddah, Saudi Arabia.

Methods

At KAUH, Jeddah, a retrospective study on 423 type 2 diabetes patients (2011-2021) was conducted, analyzing clinical outcomes, including BMI and blood glucose levels, using IBM SPSS Statistics for Windows, Version 23 (Released 2015; IBM Corp., Armonk, NY, USA). Ethical approval was granted by the Institutional Review Board, ensuring compliance with Good Clinical Practice (GCP) Guidelines.

Results

In this study, 423 diabetic patients with an average body mass index (BMI) of 29.12 kg/m² and ages ranging from 24 to 104 years (mean, 63.67) were primarily male, with 216 patients accounting for 51.1%. A total of 227 patients (53.7%) were aged 60-79 years. Most participants, 400 patients (94.6%), had average bone mineral density (BMD), while 161 patients (38.1%) were classified as obese. Among those with abnormal BMD, 23 patients (5.4%); 12 (2.8%) were identified as having osteopenia, and 11 (2.6%) were diagnosed with osteoporosis. Glycemic management and bone health indicators were examined in the lab, along with comprehensive BMD and T/Z scores.

Conclusion

This study enhances our understanding of the relationship between type 2 diabetes and bone health, emphasizing the importance of individualized evaluations for high-risk populations. Further research is needed to explore the complex interplay between diabetes, bone health, and demographic factors, to inform more effective preventive and therapeutic strategies.

## Introduction

Osteoporosis is a condition characterized by reduced bone density and microarchitecture deterioration, leading to an increased risk of fractures and significant morbidity. Osteopenia shares a similar mechanism, but the reduction in bone mineral mass is less severe than in osteoporosis. As the elderly population rises, so does the prevalence of osteoporosis, resulting in increased healthcare costs, alongside the high morbidity and mortality associated with fractures in osteoporotic bones. Postmenopausal women, who experience estrogen loss, a hormone essential for regulating bone turnover, are particularly vulnerable. Dual X-ray absorptiometry is the most commonly used diagnostic method for osteoporosis. However, as a silent disease, it is often diagnosed only after a fracture occurs [1-3].

A recent study in Saudi Arabia reported that among individuals aged 50-79, the prevalence of osteoporosis is 34% in women and 30.7% in men, with these rates expected to rise over time [4]. To determine whether participants in this study had osteoporosis or osteopenia, we used bone mineral density (BMD) scan results. BMD results are reported as a T-score, which compares an individual’s BMD to that of a young, healthy reference population, and a Z-score, which compares it to an age-, gender-, and ethnicity-matched population. A T-score of -1 or above is normal, between -1 and -2.5 indicates osteopenia, and below -2.5 indicates osteoporosis [5].

Diabetes, a long-term metabolic disease characterized by hyperglycemia, affects multiple systems, including the skeletal system [1]. It can be classified into two main categories: insulin-dependent diabetes mellitus (type 1) and non-insulin-dependent diabetes mellitus (type 2) [6]. Osteoporotic fractures are up to 12 times more likely in diabetic patients, emphasizing the importance of preventing pathological fractures in this population. A retrospective study identified osteoporotic alterations in 40%-50% of diabetic patients, highlighting the need for targeted screening [1]. Similarly, a cohort study in Taiwan revealed that individuals with type 2 diabetes have a 1.5 to 2 times higher risk of developing osteoporosis compared to non-diabetic populations [2]. The relationship between diabetes and BMD remains unclear, as studies have shown conflicting results. According to research, Saudi Arabia has the highest number of diabetic patients in the Middle East, with the prevalence rate expected to continue rising [7,8].

With the increasing prevalence of both diseases and their unclear relationship, it is important to explore this connection further. This study hypothesizes that patients with type 2 diabetes have a higher prevalence of osteoporosis and osteopenia compared to the general population. It will assess this relationship in patients at King Abdulaziz University Hospital (KAUH), in Jeddah, Saudi Arabia.

## Materials and methods

At KAUH, a tertiary care facility in Jeddah, a retrospective record review was carried out on a single-center basis.

The KAUH Institutional Review Board approved this study (Ref: 562-22). All procedures adhered to the ethical standards of the relevant committee and the principles outlined in the Good Clinical Practice (GCP) Guidelines. Because the study was retrospective, informed consent was not required. We collected and analyzed the data of 551 patients diagnosed with type 2 diabetes between 2011 and 2021.

The inclusion criteria for this study were as follows: (1) patients diagnosed with type 2 diabetes mellitus, and (2) individuals who underwent BMD testing at KAUH.

The exclusion criteria for this study were as follows: (1) patients with type 1 diabetes, (2) non-diabetic patients, (3) patients with incomplete or missing records, (4) patients who have not had a BMD scan, and (5) patients who are not treated at KAUH.

The number of patients who fulfilled the inclusion criteria was 423, and we included patients of all ages diagnosed with type 2 diabetes. Patients with no documentation of laboratory investigations or with type 1 diabetes were excluded.

The sample size was retrospectively justified using a prevalence of osteoporosis of 34% in postmenopausal women and 30.7% in men, as reported in previous studies. With a 95% confidence level and a 5% margin of error, the required sample size was calculated to be approximately 323 participants. Our study included 423 participants, exceeding this requirement and ensuring sufficient statistical power to detect significant associations.

Clinical data

The clinical data of our included patients were acquired via the hospital records. Data that was retrieved included age, gender, diagnosis of type 2 diabetes, diagnosis of osteoporosis, diagnosis of osteopenia, whether the patient was female and menopausal (>50), smoking history, history of alcohol abuse, body mass index (BMI), random blood glucose levels at presentation, glycated hemoglobin A1c (HbA1c) levels, serum calcium levels (mmol/L), serum phosphate levels (mmol/L), use of glucocorticoids, history of surgery due to previous fractures, parathyroid hormone (PTH) levels, bone alkaline phosphatase (BALP) levels, serum 25-hydroxy vitamin D (25(OH)D) (nmol/L) levels, serum thyroid-stimulating hormone (TSH) levels, and, finally, BMD scan distribution.

Statistical analysis

Data were expressed as mean ± standard deviation (minimal-maximum) and median (25th and 75th percentiles) for parametric data, and frequency (%) for categorical data. Analysis was performed using IBM SPSS Statistics for Windows, Version 23 (Released 2015; IBM Corp., Armonk, NY, USA). The Shapiro-Wilk test was employed to determine the normality of parametric value distributions. The Mann-Whitney test was utilized to compare groups of abnormally distributed parametric data (all laboratory investigations), and the Pearson Chi-square test and Fisher's Exact test were used for comparison of categorical data, as appropriate. A p-value of <0.05 was considered statistically significant.

Regressional analysis

A multivariable logistic regression analysis was conducted to assess the relationship between abnormal BMD and key variables, including age, gender, and BMI. The analysis aimed to adjust for potential confounding factors and estimate the independent effect of each variable on BMD status (normal vs. osteopenia/osteoporosis). Odds ratios (ORs) with 95% confidence intervals were calculated using IBM SPSS Statistics for Windows, Version 23.

## Results

Table 1 shows the demographic characteristics of 423 diabetic patients included in this study. The age of the patients ranged from 24 to 104 years, with a mean of 63.67 years. Most of the included patients, 227 (53.7%), were in the age group from 60 to 79 years, followed by 119 (28.1%) in the 40-59 years group, 55 (13.0%) aged ≥80 years, and 22 (5.2%) in the 24-39 years group. The majority of included patients were male (216, or 51.1%), compared to female (207, or 48.9%).

**Table 1:** Demographic characteristics of all patients (n = 423) Data were expressed as mean ± standard deviation (minimum-maximum) or frequency (%) as appropriate.

Characteristics	All patients
Age (years)	63.67 ± 13.22 (24-104)
24-39 years	22 (5.2%)
40-59 years	119 (28.1%)
60-79 years	227 (53.7%)
≥80 years	55 (13.0%)
Gender	
Male	216 (51.1%)
Female	207 (48.9%)
Body mass index (BMI) (kg/m^2^)	29.12 ± 6.55 (15.62-75.75)
Underweight (<18.5 kg/m^2^)	7 (1.7%)
Normal weight (18.5-24.9 kg/m^2^)	111 (26.2%)
Overweight (25-29.9 kg/m^2^)	144 (34.0%)
Obesity (≥30 kg/m^2^)	161 (38.1%)
Risk factors	
Postmenopausal	168 (39.7%)
Smoking	94 (22.2%)
Alcohol consumption	3 (0.7%)
Use of glucocorticoids	207 (48.9%)
History of surgery due to previous fracture	17 (4.0%)

BMI ranged from 15.62 to 75.75 kg/m², with a mean of 29.12 kg/m². Among the patients, 161 (38.1%) were classified as obese, 144 (34.0%) as overweight, 111 (26.2%) as normal weight, and seven (1.7%) as underweight. Of the included patients, 168 (39.7%) were postmenopausal, 94 (22.2%) were cigarette smokers, three (0.7%) consumed alcohol, 207 (48.9%) used steroids, and 17 (4.0%) had a history of previous fractures.

Table 2 shows the laboratory investigations performed for all patients, including glycemic control (serum blood glucose levels and HbA1C), serum calcium and phosphorus levels, PTH, BALP, 25(OH)D, and TSH levels.

**Table 2:** Laboratory investigations done for all patients (n = 423) Data were expressed as mean ± standard deviation (minimum-maximum) and median (25th-75th percentile).

Investigations	Mean ± SD (minimum-maximum)	Median (25th-75th percentile)
Blood glucose (mmol/L)	10.92 ± 5.03 (3.40-36.60)	9.80 (7.20-13.75)
Glycated hemoglobin A1c (HbA1c) (%)	8.11 ± 2.09 (4.10-15.30)	7.70 (6.50-9.35)
Serum calcium (mmol/L)	2.17 ± 0.24 (1.38-4.34)	2.18 (2.07-2.27)
Serum phosphate (mmol/L)	1.19 ± 0.48 (0.34-5.37)	1.09 (0.92-1.33)
Parathyroid hormone (PTH) (pg/mL)	17.11 ± 17.31 (1.10-97.68)	11.18 (6.65-21.07)
Bone alkaline phosphatase (BALP) (µg/L)	118.69 ± 90.74 (31.00-564.00)	91.00 (67.00-131.00)
25-hydroxy vitamin D (25(OH)D) (nmol/L)	49.64 ± 32.81 (7.50-194.50)	41.59 (26.58-61.78)
Thyroid-stimulating hormone (TSH) (mlU/L)	2.70 ± 2.94 (0.01-19.90)	1.91 (1.08-3.09)

BMD was 0.74 ± 0.18 g/cm², T-scores were -1.64 ± 1.39, and Z-scores were 0.03 ± 1.09, -0.08 ± 1.41, and -0.335 ± 1.148. BMD was normal in 400 patients (94.6%), while abnormal BMD was found in 23 patients (5.4%), among whom 12 patients (2.8%) had osteopenia and 11 patients (2.6%) had osteoporosis (Table 3).

**Table 3:** Bone mineral density of patients Data were expressed as mean ± standard deviation (minimum-maximum) and frequency (%) as appropriate.

Characteristics	Value
Bone mineral density (BMD) (g/cm²)	0.74 ± 0.18 (0.354 to 1.139)
T-score	-1.64 ± 1.39 (-5.100 to 0.800)
Z-score	-0.335 ± 1.148 (-2.60 to 1.600)
Bone mineral density (BMD) status	
Normal	400 (94.6%)
Abnormal	23 (5.4%)
Osteopenia	12 (2.8%)
Osteoporosis	11 (2.6%)

Table 4 shows the demographic characteristics of diabetic patients with normal and abnormal bone status (osteoporosis and osteopenia). Abnormal BMD was significantly higher than normal BMD in females (19 patients, or 82.6% vs. 188 patients, or 47.0%; p < 0.001), obese individuals (14 patients, or 60.9% vs. 147 patients, or 36.8%; p = 0.013), postmenopausal patients (18 patients, or 78.3% vs. 150 patients, or 37.5%; p < 0.0001), and glucocorticoid users (18 patients, or 78.3% vs. 186 patients, or 47.2%; p = 0.003).

**Table 4:** Demographic characteristics of patients with normal and abnormal BMD Data were expressed as frequency (%) as appropriate. A comparison was made between groups by the Pearson Chi-square test and Fisher's Exact test as appropriate. BMD: bone mineral density

Characteristics	Normal BMD (n = 400)	Abnormal BMD (n = 23)	Significance
Age groups			p = 0.425
24-39 years	22 (5.5%)	-	-
40-59 years	115 (28.7%)	4 (17.4%)	
60-79 years	211 (52.8%)	16 (69.6%)	
≥80 years	52 (13.0%)	3 (13.0%)	
Gender			p < 0.001
Male	212 (53.0%)	4 (17.4%)	-
Female	188 (47.0%)	19 (82.6%)	
Body mass index (BMI) groups			p = 0.031
Underweight (<18.5 kg/m^2^)	7 (1.8%)	-	-
Normal weight (18.5-24.9 kg/m^2^)	110 (27.5%)	1 (4.3%)	
Overweight (25-29.9 kg/m^2^)	136 (34.0%)	8 (34.8%)	
Obesity (≥30 kg/m^2^)	147 (36.8%)	14 (60.9%)	
Risk factors			
Postmenopausal	150 (37.5%)	18 (78.3%)	p < 0.0001
Smoking	92 (23.0%)	2 (8.7%)	p = 0.127
Use of glucocorticoids	6 (75.0%)	-	-
History of alcohol consumption	3 (0.8%)	-	p = 0.845
Use of glucocorticoids	186 (47.2%)	18 (78.3%)	p = 0.003
History of surgery due to previous fracture	14 (3.5%)	3 (13.0%)	p = 0.058

Table 5 shows the laboratory investigations of diabetic patients with normal and abnormal bone status (osteoporosis and osteopenia). BALP was significantly lower in patients with abnormal BMD (92.67 ± 86.48 µg/L) than in those with normal BMD (121.11 ± 90.95 µg/L, p = 0.012). Meanwhile, 25(OH)D was significantly higher in patients with abnormal BMD (65.01 ± 34.21 nmol/L) compared to those with normal BMD (48.12 ± 32.36 nmol/L, p = 0.016).

**Table 5:** Laboratory investigations of patients with normal and abnormal BMD Data were expressed as mean ± SD and median (25th-75th percentile). Comparison between groups was made by the Mann-Whitney test, as the data were abnormally distributed. BMD: bone mineral density

Investigations	Normal BMD (n = 400)	Abnormal BMD (n = 23)	Significance
Blood glucose (mmol/L)	10.98 ± 5.06	9.77 ± 4.38	p = 0.273
Median (25th-75th percentile)	9.80 (7.20-13.80)	8.60 (6.55-11.50)	
Glycated hemoglobin A1c (HbA1c) (%)	8.13 ± 2.10	7.69 ± 1.88	p = 0.324
Median (25th-75th percentile)	7.70 (6.50-9.40)	7.10 (6.30-9.10)	
Serum calcium (mmol/L)	2.16 ± 0.23	2.24 ± 0.26	p = 0.120
Median (25th-75th percentile)	2.17 (2.07-2.27)	2.25 (2.08-2.34)	
Serum phosphate (mmol/L)	1.19 ± 0.48	1.07 ± 0.35	p = 0.312
Median (25th-75th percentile)	1.10 (0.92-1.32)	1.01 (0.86-1.37)	
Parathyroid hormone (PTH) (pg/mL)	17.69 ± 17.73	12.30 ± 12.85	p = 0.174
Median (25th-75th percentile)	11.33 (6.86-21.37)	9.47 (2.35-20.06)	
Bone alkaline phosphatase (BALP) (µg/L)	121.11 ± 90.95	92.67 ± 86.48	p = 0.012
Median (25th-75th percentile)	95.00 (69.00-136.00)	80.00 (50.00-102.50)	
25-hydroxyvitamin D (25(OH)D) (nmol/L)	48.12 ± 32.36	65.01 ± 344.21	p = 0.016
Median (25th-75th percentile)	40.40 (25.98-59.63)	63.80 (38.53-91.75)	
Thyroid-stimulating hormone (TSH) (mlU/L)	2.74 ± 2.95	2.23 ± 2.86	p = 0.212
Median (25th-75th percentile)	1.93 (1.07-3.30)	1.52 (0.99-2.80)	

The logistic regression analysis revealed that gender was a significant predictor of abnormal BMD (OR = 5.10, 95% CI: 1.67-15.60, p = 0.004). Females had significantly higher odds of having abnormal BMD compared to males. Age showed a weak positive association with abnormal BMD (OR = 1.03, 95% CI: 0.99-1.06, p = 0.105), but this was not statistically significant. Similarly, BMI was not significantly associated with abnormal BMD (OR = 1.02, 95% CI: 0.97-1.08, p = 0.373).

## Discussion

Demographics of our study

In line with worldwide trends, our research revealed an increased incidence of diabetes in older age groups, particularly among the 227 patients (53.7%) in the 60-79 age group. The focus on the 168 postmenopausal women (39.7%) in our findings is particularly significant. Hormonal changes after menopause, which impact body composition and insulin sensitivity, make this population more vulnerable to diabetes. These developments underscore the importance of targeted diabetes prevention and control measures, especially for postmenopausal populations. Raška et al. (2017) highlighted a 25% prevalence of osteoporosis among postmenopausal patients with type 2 diabetes, noting their increased likelihood of low-trauma vertebral and nonvertebral fractures. This finding emphasizes the need for bone health screening in this group. Our study results revealed that postmenopausal women with type 2 diabetes did not exhibit significantly different BMDs, aligning with existing knowledge on the effects of diabetes on bone health. However, lower osteocalcin levels and higher sclerostin levels were observed in diabetic patients, possibly due to decreased physical activity, which could reduce bone remodeling potential and quality [9].

A multivariable logistic regression analysis, controlling for age, gender, BMI, and glucocorticoid use, identified gender as a significant predictor of abnormal BMD, with females exhibiting substantially higher odds compared to males. While age, BMI, and glucocorticoid use were positively associated with abnormal BMD, these associations were not statistically significant. These findings underscore the importance of gender-specific strategies in managing bone health in diabetic patients.

BMI and bone health in diabetic patients

The mean BMI of our patients was 29.12 kg/m², with 161 patients (38.1%) classified as obese, highlighting the well-established link between type 2 diabetes and obesity. Raška et al. (2017) similarly noted that reduced physical activity, as evidenced by higher sclerostin levels in less active diabetic women, could adversely impact bone health [9].

BMD in diabetic patients

Our data revealed that individuals with abnormal BMD were more likely to exhibit risk factors, with 18 patients (78.3%) being postmenopausal and 18 patients (78.3%) having a history of glucocorticoid use, both of which contributed significantly to bone mass deterioration. Laboratory findings showed lower levels of BALP and higher levels of 25(OH)D in individuals with abnormal BMD, suggesting unique metabolic alterations influencing bone health. Wen et al. (2021) highlighted the role of bone turnover markers, such as BALP, in identifying impaired bone remodeling and increased fracture risk in type 2 diabetes. These findings underscore the need for further research on the interplay between abnormal BMD, metabolic factors, and bone health in diabetic populations [10].

Among the 423 type 2 diabetic participants, 12 patients (2.8%) had osteopenia, and 11 patients (2.6%) had osteoporosis, indicating a lower prevalence of bone mass degeneration compared to previous reports. Huang et al. (2022) identified age, female gender, BMI, low-density lipoprotein (LDL) cholesterol, serum uric acid, and glycemic variability (assessed by Mean Amplitude of Glycemic Excursions, or MAGE) as key markers contributing to osteoporosis in diabetics. Furthermore, the Trabecular Bone Score (TBS), as demonstrated by Shevroja et al. (2021), evaluates bone microarchitecture independently of BMD and predicts fracture risk. Incorporating TBS with quantitative BMD testing provides a more comprehensive assessment of bone health and fracture risk in diabetic patients. These findings highlight the need for a holistic evaluation of bone health in type 2 diabetes, accounting for both bone density and quality, which are influenced by various metabolic factors [11,12].

Additionally, a study using data from the Korea National Health and Nutrition Examination Survey revealed that males with prediabetes and diabetes exhibited higher mean BMD across all assessed locations compared to controls. However, a longer diabetes duration was associated with lower BMD in the whole hip and femoral neck areas. This indicates that while diabetes may initially be associated with higher BMD, its long-term effects could negatively impact bone health, particularly in specific regions like the femoral neck [13].

Starup-Linde et al. (2016) further examined bone density and structure in individuals with type 1 and type 2 diabetes, reporting higher BMD in type 2 diabetics compared to type 1 at key locations such as the hip, femur, and spine. However, only hip differences remained significant in multivariate-adjusted models. Additionally, the study found that type 2 diabetics exhibited higher bone tissue stiffness at the tibia. Among type 1 diabetics, sclerostin levels negatively correlated with fractures, suggesting that sclerostin may serve as an independent fracture risk marker. These findings enhance our understanding of how diabetes type and duration influence bone health. They emphasize the importance of considering bone quality, metabolic parameters, and TBS, alongside BMD, when evaluating fracture risk in diabetic patients [14].

Laboratory investigations and diabetes management

Our research on hormone levels and glycemic control in managing type 2 diabetes aligns with the findings of Huang et al. (2022), which emphasize the relationship between osteoporosis and glycemic variability, a factor often overlooked in diabetes management. Patients with osteoporosis and type 2 diabetes exhibited higher average blood glucose levels and greater fluctuations, with key metrics such as time in range and MAGE varying significantly. These findings suggest that glycemic variability and blood glucose fluctuations may be as critical as average glucose levels in assessing bone health in diabetic patients [11].

Data from Sheu et al. (2022) highlighted the link between type 2 diabetes and skeletal fragility, even when BMD is preserved. This raises concerns about the adequacy of existing fracture risk calculators, which primarily focus on BMD, in predicting fracture risk for diabetic individuals. The findings underscore the need for a holistic approach to diabetes management, incorporating comprehensive bone health assessments alongside glucose control [15].

Implications for clinical practice

The association between osteoporosis and elevated glycemic variability in type 2 diabetes patients, as demonstrated by Leslie et al. (2012) [16], highlights the importance of managing both average blood glucose levels and fluctuations to maintain bone health. This is particularly relevant, given the recognition of diabetes as an independent risk factor for fractures.

Diabetes-related changes in bone composition and structural characteristics may explain the paradox of normal or elevated BMD in older individuals with type 2 diabetes, who nevertheless face increased fracture risk. Factors such as impaired bone turnover and an excessively glycated collagen matrix can compromise the biomechanical strength of bones in this population. Current clinical tools, such as FRAX (Fracture Risk Assessment Tool), may be inadequate for predicting fracture risk in diabetic patients, as they rely heavily on BMD.

These findings underscore the need for a more nuanced approach to diabetes management that accounts for its broader effects on bone health. This includes adopting more comprehensive FRAX tools tailored to older adults with type 2 diabetes. Managing glycemic variability and addressing diabetes-related alterations in bone quality should drive changes in how osteoporosis is evaluated and treated in diabetic patients [16].

Limitations and future research

This study’s retrospective design introduces potential selection bias, and the low number of patients diagnosed with osteoporosis and osteopenia is a notable limitation. This may be due to the small proportion of patients who underwent BMD scans, which could lead to an underestimation of the true prevalence of bone mass degeneration in our cohort.

Additionally, the study did not account for bone-related comorbidities or the long-term use of medications, except for steroid therapy, that could influence bone health. Future research should include these variables to provide a more comprehensive analysis. Similarly, detailed data on the use of antidiabetic or osteoporosis medications, which may affect BMD, were not included in this study and should be addressed in future investigations.

A significant proportion of the study population was on long-term steroid therapy, potentially confounding the relationship between type 2 diabetes and BMD. Prospective studies should stratify patients based on corticosteroid use to better evaluate the independent effects of diabetes on bone health.

One of the limitations of this study is the absence of a healthy control group for comparison. While the primary objective was to assess the prevalence of osteoporosis among patients with type 2 diabetes, the lack of a control group limits the ability to directly evaluate the relative risk of osteoporosis in diabetic versus non-diabetic populations. Future studies should aim to include a control group to better establish the relationship between diabetes and osteoporosis.

Another limitation of this study is the absence of comprehensive fracture data due to our hospital’s non-trauma center status. Future studies might be conducted at a trauma center to better establish fracture risk in diabetes.

Future research should also explore the complex interplay among diabetes, BMI, and bone health, with a particular focus on specific populations, such as postmenopausal women. Increasing the number of participants undergoing BMD assessments will be critical for providing a more accurate estimate of osteoporosis and osteopenia prevalence in diabetic populations.

## Conclusions

This study contributes to a better understanding of the complex relationship between type 2 diabetes, BMI, and bone health. While routine BMD assessments may not be necessary for all diabetic patients, personalized evaluations remain essential, particularly for individuals at higher risk, such as postmenopausal women. These targeted assessments can help identify patients who may benefit from early interventions to reduce the risk of fractures and other complications.

Furthermore, this research emphasizes the need for additional studies to explore the intricate connections between diabetes, bone health, and demographic factors. Such investigations will provide deeper insights into the mechanisms underlying these associations and support the development of more tailored preventive and therapeutic strategies to improve outcomes for diabetic patients.
